# FBA Ecological Guild: Trio of *Firmicutes-Bacteroidetes* Alliance against *Actinobacteria* in Human Oral Microbiome

**DOI:** 10.1038/s41598-019-56561-1

**Published:** 2020-01-14

**Authors:** Wendy Li, Zhanshan (Sam) Ma

**Affiliations:** 1 Computational Biology and Medical Ecology Lab, State Key Laboratory of Genetic Resources and Evolution, Kunming Institute of Zoology, Chinese Academy of Sciences, Kunming, China; 2 Center for Excellence in Animal Evolution and Genetics, Chinese Academy of Sciences, Kunming, 650223 China; 3 Kunming College of Life Sciences, University of Chinese Academy of Sciences, Kunming, China

**Keywords:** Network topology, Microbial ecology, Microbiome

## Abstract

In a pioneering study, Zaura *et al*. (2009) found that majority of oral microbes fall within the five phyla including, *Firmicutes, Proteobacteria, Actinobacteria, Bacteroidetes and Fusobacteria*. Subsequent studies further identified a set of microbes that were commonly shared among unrelated individuals (*i.e*., core). However, these existing studies may have not been designed to investigate the interactions among various *core* species. Here by harnessing the power of ecological network analysis, we identified some important *ecological guilds* in the form of network clusters. In particular, we found that the strongest cluster is an alliance between *Firmicutes* and *Bacteroidetes* against *Actinobacteria* (FBA-guild). Within the guild, we further identified two sub-guilds, the *Actinobacteria-dominant* sub-guild (ASG) and *Firmicutes*-dominant allied with *Bacteroidetes* sub-guild (FBSG). Furthermore, we identified so-termed *guard* nodes in both sub-guilds, and their role may be to inhibit the peer sub-guild given they held competitive interactions only with the outside nodes only but held cooperative interactions only with the internal nodes, which we termed *civilian* nodes given that they only held cooperative interactions. We postulated that FBA-guild might be to do with protection of oral health against some opportunistic pathogens from *Corynebacterium* and *Actinomyces*, the two major genera of *Actinobacteria* (target of FB alliance).

## Introduction

The investigation of the human oral microbiome is among the earliest studies of the human microbiome. For example, as early as in the 1990s, scientists and clinicians have already resorted to ecological theories to interpret the etiology of periodontitis^1,2^. The oral cavity is a complex ecosystem comprised of many habitats, such as tongue, palates, cheeks, teeth and gingival sulcus. These inter-connected intra-oral habitats may have different microbial profiles, and the whole oral microbiome is therefore a meta-microbial community. The oral microbiome plays a critical role in maintaining our oral health, and its dysbiosis can lead to oral diseases such as dental caries, gingivitis and periodontitis^3–9^. In addition, recent studies have revealed that oral microbiome may also be associated with many systemic diseases including atherosclerosis^10^, gastrointestinal cancer^11^, inflammatory bowel disease^12^ and diabetes^13^.

The composition of oral microbiome is influenced by host genetics^14^, and fluctuates with host health status and lifestyle-related factors^15^, which could lead to great inter-subject heterogeneity. For example, diet is one of the major factors that disturb the balance of oral microbiota. Adler *et al*. (2013) showed that, compared with hunter-gatherer diet, carbohydrate-rich farming diet (or modern diet) lead to the modern oral microbiota with low biodiversity and the dominance of *cariogenic* bacteria^16^. Moreover, some studies have found that smoking altered the structure of oral microbiome, which increase the risk for periodontitis^17,18^. Health status and habitats may directly or indirectly influence the factors of host intra-oral environment, such as pH and iron, that has been reported to have significant influences on the oral microbiota^19^. However, recent studies (*e.g*., He *et al*. 2014, Belstrom *et al*. 2016) have also suggested that the oral microbiome is relatively stable and resistant to species invasions^20,21^. The maintenance factors of oral microbiota, as Zaura *et al*. (2014) reviewed, include host-derived and microbe-derived, in which the host immune system plays a key role on the homeostasis of oral microbial community^22^. In the meantime, oral micro-ecosystem may also help to improve and perfect the host immune system. The invasion-resistance and inter-species interactions as microbe-derived factors are also important to maintain the stability of oral microbiome^22^.

A primary mission of NIH-HMP (human microbiome project) was to answer the question whether there is a core set of species in the human microbiome, and the studies of human oral microbiome were set with a similar goal^18,23–26^. In a seminal study on the oral microbiome, Zaura *et al*. (2009) defined the oral core microbiome as the *commonly shared unique sequences* (phylotypes) among unrelated individuals^24^. Through multi-site studies of the healthy oral microbiome, Zaura *et al*. (2009) detected over 500 species in each individual oral microbial community, and the majority of taxa fall within the five phyla including *Firmicutes, Proteobacteria, Actinobacteria, Bacteroidetes* and *Fusobacteria*^24^. Other studies have also identified the presence of a common core microbiome in the human oral cavity, which is generally defined as the phylotypes or operational taxonomic units (OTUs), in a specific healthy habitat, that are shared among the vast majority of humans^24,27–29^. Nevertheless, the pursuing of core microbiome turned out to be more elusive than initially^30–32^. As Zaura *et al*. suggested, the studies of oral microbiome should be shifted to functional approaches such as metabolism, and more advanced topics such as the interactions between fungi and bacteria, host environment and microbiota, as well as the *inter-species interactions* should be paid to more^33–35^.

The objective of this study is to further investigate the *inter-species interactions* in the core of oral microbiome by detecting and analyzing the important clusters (or ecological *guilds*) in the oral microbiome network by reanalyzing the datasets originally published by Zaura *et al*. (2009) for investigating core of the oral microbiome^24^. An in-depth study of the core oral microbiota should be critical for understanding the structure and stability mechanism of oral microbiome, which can also be significant for investigating the etiology of oral diseases.

## Materials and Methods

### The oral microbiome datasets

The 16S ribosomal RNA datasets of the human oral microbiome analyzed in this report were first reported in Zaura *et al*.^24^. The oral samples were selected from several sites of three healthy male adults, including dental surfaces of upper incisor and upper molar, mucosa of cheek, hard palate and tongue surface, and saliva. A total of 29 oral microbiome samples from three healthy individuals were collected and sequenced with 16s-rRNA amplicon sequencing technology. Each individual were sampled at 9 or 10 oral sites, and on average, 6315 unique sequences were obtained for each sample. There were 818 OTUs (operational taxonomic units) identified at 97% similarity level. Each OTU was labeled after their lowest annotated taxonomic level and a unique number (such as *Corynebacterium*_767).

Although the number of individuals is relatively small, the sampled sites from each individual (9–10) as well as the reads per sample (6315 on average) are sufficiently large to enable our network analysis for investigating core oral microbiota. In particular, we take advantages of the findings and insights on the oral microbial core obtained from this same datasets by the original scientists^24^.

### The network analysis approach

We adopted standard approach for correlation network analysis to construct and analyze the human oral microbiome network with the 16s-rRNA datasets originally reported by Zaura *et al*.^24,36–39^. To reduce the noise effect of the OTUs with extremely low abundance and potentially spurious OTU reads, we filtered out the OTUs whose total reads in all 29 samples were less than 30, *i.e*., approximately one read per sample, equivalent to removing the so-called *singleton*, which is a common practice in ecological analysis. A total of 347 OTUs remain after the filtering operation, and their abundances were utilized to construct the species correlation network, based on Spearman’s rank correlation coefficient (*R*). The correlation relationships with |*R*| ≥ 0.6 and *p*-value ≤ 0.05 (significance level) were set as criteria for selecting network edges (links).

Cytoscape software (Version 2.8.3) was used to visualize the network graphs and MCODE plug-in for Cytoscape for detecting network clusters (modules)^36,40,41^. MCODE (Molecular Complex Detection) is a graph-theoretic clustering algorithm, which was first introduced by Bader *et al*. (2003) to identify molecular complexes in large protein interaction networks^41^. The molecular complexes in a protein network can be considered as the locally dense regions or clusters in a graph. The core algorithm of the MCODE is to detect the clusters of vertex weighted based on the local neighborhood density or cliquishness, which can be measured by the clustering coefficient, *C*_*i*_,

$${C}_{i}=2n/{k}_{i}({k}_{i}-1)$$

where *k*_*i*_ is the number of neighborhood vertices of vertex *i*, and *n* is the number of edges (links) in the neighborhood. There are three main steps in the MCODE algorithms. First, MCODE weights all vertices based on their local neighborhood density, generating the so-termed vertex weighted graph (VWG). Next, the locally highest weighted vertex in the VWG will be set as a seed for a candidate cluster, and the cluster will be isolated by outwardly traversing from the seed to find all the vertices whose weights are within a given threshold. The third step is post-processing to filter vertices in the candidate cluster according to the given parameter sets. The detail interpretation of the algorithm is referred to Bader *et al*.^41^.

In addition, iGraph R-package was utilized for computing the network properties^42^. We also identified the P/N (positive to negative links) ratio in the network, which is a network property proposed by Ma (2017) to measure the balance between cooperative and competitive interactions in the microbiome^43^.

## Results and Discussion

### Basic network properties

The oral microbiome network we reconstructed contained 335 nodes (OTUs) and 4335 links (3692 positive links and 643 negative links). Table 1 lists the basic network properties. As shown in Table 1, the ratio of positive to negative correlation relationships is approximately 5.7, which suggests that the oral microbiome network is predominantly cooperative^43^. Since these network properties do not offer much intuitive insights on the oral microbiome network, we focus on the detection of network clusters, which are equivalent to the *guild* in ecological community, through which we expect to deepen our understanding on the critical species interactions in the oral microbiome and to further shed light on the structure and functions of core oral microbiota or guilds.

**Table 1:** The basic properties of the oral microbiome network.

Num. of Nodes	Num. of Edges	Average Degree	Avg. Local Cluster Coefficient	Diameter	Average Path Length	Connected Components	Network Density	Network Modularity	Num. of Communities	Ratio of Positive to Negative
335	4335	25.881	0.509	10	2.784	1	0.077	0.379	64	5.742

### FBA Guild—*Firmicutes-Bacteroidetes* ally against *Actinobacteria*

An ecological guild can be defined as a group of species that exploit the same resources or exploit different resources in related manners^44^. Guild members could be competing for resources and hence hold negative correlation relationships in their abundances in the species correlation network of the oral microbiome. They may also *cooperatively* exploit other resources and therefore hold positive correlation relationships. Although rigorously defining and identifying microbial guilds can be rather challenging at this stage of human microbiome research mainly because functional studies on the microbiome are still scarce, we believe that the following exploration for bacterial guilds through network cluster detection technique is the best we can perform in order to deepen our understanding on the interspecific interactions and to further shed light on the structure and functions of core oral microbes.

The technique we use for detecting network clusters (modules or ecological guilds) is the MCODE plug-in for Cytoscape^40,41^. Table 2 lists the 11 clusters we detected with MCODE, including the cluster number, cluster score, number of nodes and number of edges for each cluster. The cluster score is a measure of the cluster density. The higher the cluster score is, and the stronger the corresponding cluster is. The strongest cluster (*i.e*., No. 1 cluster in Table 2) contains 55 nodes and 648 edges, which is nearly 1/6 of all OTUs in the oral microbiome network.

**Table 2:** The network clusters (bacterial guilds) detected in the oral microbiome network.

Cluster No.	Score	Nodes	Edges
1	11.78	55	648
2	7.04	48	338
3	4.81	26	125
4	2.63	32	84
5	2.28	18	41
6	2.10	21	44
7	2	5	10
8	1.88	8	15
9	1	3	3
10	1	3	3
11	1	3	3

**Figure 1:**
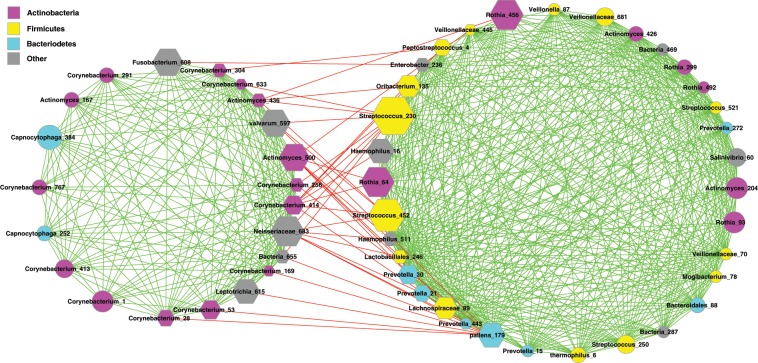
The strongest cluster (FBA cluster) in the healthy oral microbiome network. Symbols used: nodes in magenta—the OTUs of *Actinobacteria* phylum, nodes in yellow—the OTUs of *Firmicutes* phylum, nodes in cyan— the OTUs of *Bacteroidetes* phylum, nodes in gray—the OTUs of other phyla; edges in green— positive correlations, edges in red—negative correlations.

The strongest cluster primarily consists of the OTUs from three phyla: *Actinobacteria*, *Firmicutes*, and *Bacteriodetes*, and we term the strongest cluster as FBA-cluster with the initials of the three phyla (Fig. 1). As shown in Tables 3, 38.2% (21 out of 55) of the OTUs in the FBA-cluster belong to *Actinobacteria*, 25.5% (14 out of 55) to *Firmicutes*, and 16.4% (9 out of 55) to *Bacteriodetes*. As further illustrated below, FBA cluster or *guild* is a trio of *Firmicutes-Bacteriodete* (*F-B*) ally against *Actinobacteria*, in which *Firmicutes* and *Bacteriodetes* hold positive links and both hold negative links with *Actinobacteria* in oral microbiome network. In other words, the F-B coalition competes against *Actinobacteria*, and each of them holds negative relationship with their common ‘enemy’.

**Table 3:** The structure (major components or sub-clusters) of FBA cluster.

Cluster	Total Num. of OTUs	*Actinobacteria*	*Firmicutes*	*Bacteroidetes*	Others
FBA-Guild	55	21 (38.2%)	14 (25.5%)	9 (16.4%)	11 (20.0%)
ASG (*Actinobacteria*-Dominant Sub-Guild)	21	14 (66.7%)	0 (0%)	2 (9.5%)	5 (23.8%)
FBSG (*Firmicutes*-Dominant Sub-Guild)	34	7 (20.6%)	14 (41.2%)	7 (20.6%)	6 (17.6%)

A broadly defined ecological guild almost always contains constituent guilds or sub-guilds, and so does our FBA guild that consists of two sub-guilds. Table 3 shows the component taxa of the two sub-guilds (sub-clusters) FBA contains. One is the *Actinobacteria*-dominant sub-guild (ASG), in which nearly 2/3 (67%) of species belong to *Actinobacteria*, and no *Firmicutes* exist and the number (only 2) of species from *Bacteroidetes* is negligible in the ASG sub-guild. Another is the *Firmicutes*-dominant sub-guild, in which more than 40% of the species are from the phylum of *Firmicutes*, and 21% are from *Bacteroidetes* in this sub-guild. Given the significant presence of *Bacteroidetes* in the second sub-guild, we term it FBSG (*Firmicutes Bacteroidete*s sub-guild).

Figure 1 illustrates the topological structure of the two sub-guilds, the left side is the ASG sub-guild and the right side is the FBSG sub-guild. We consider FBSG sub-guild as an ‘alliance’ between *Firmicutes* and *Bacteroidetes* against ASG sub-guild. Our justifications include: (*i*) all interactions between *F* & *B* are cooperative, as illustrated in the all positive relationships in the FBSG sub-guild (green edge in the left sub-cluster); (*ii*) all interactions between the FBSG and ASG sub-guilds are competitive, as illustrated in the all negative relationships between the two sub-clusters (the intermediate red edges); (*iii*) the numbers of members (network nodes) in both sub-guilds (*i.e*., 21 *A* in ASG *vs*. 14 *F* + *7B* in FBSG) are also on a par with each other.

**Figure 2:**
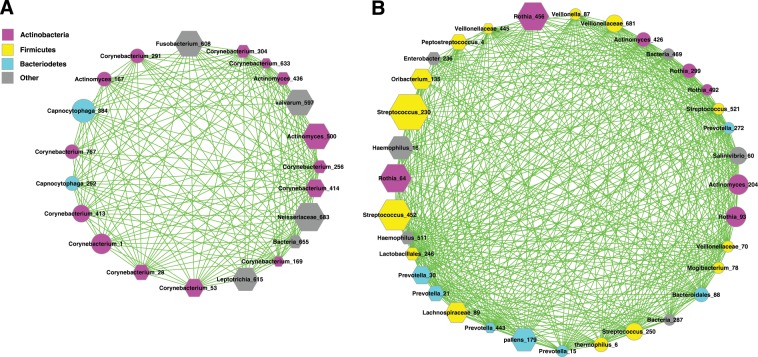
(**A**) The ASG sub-guild (*Actinobacteria*-dominant sub-guild). Symbols used: nodes colored in magenta—the OTUs of *Actinobacteria* phylum, nodes in cyan—the OTUs of *Bacteroidetes* phylum, nodes in gray—the OTUs of other phyla; edges in green—positive interactions; no negative links existed here. (**B**) The FBSG sub-guild (*Firmicutes-*dominant with *Bacteroidetes* ally sub-guild). Symbols used: nodes colored in magenta—the OTUs of *Actinobacteria* phylum, nodes in yellow—the OTUs of *Firmicutes* phylum, nodes in cyan—the OTUs of *Bacteroidetes* phylum, nodes in gray—the OTUs of other phyla, edges in green—positive correlations; no negative links existed here.

**Figure 3:**
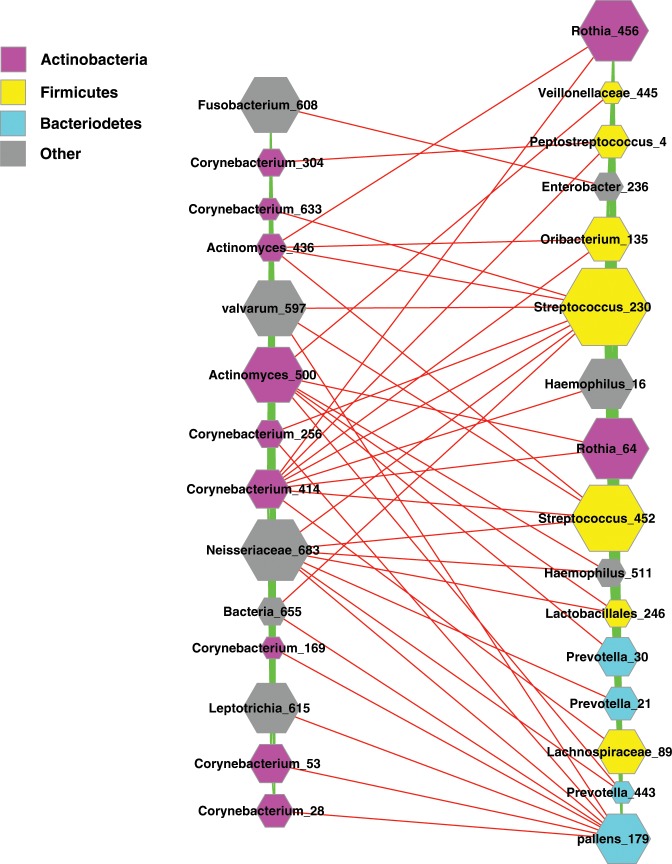
The negative relationships between the two sub-guilds of the FBA guild. Symbols used: nodes in magenta—the OTUs of *Actinobacteria* phylum, nodes in yellow—the OTUs of *Firmicutes* phylum, nodes in cyan—the OTUs of *Bacteroidetes* phylum, nodes in gray— the OTUs of other phyla; edges in green—positive correlations, edges in red—negative correlations.

Figure 2A,B were drawn to facilitate the visualization of the relationships mentioned above by deconstructing graph (Fig. 1) of FBA cluster into two regional blocks. Figure 2A shows the ASG sub-guild, *i.e*., the left block in Fig. 1. Figure 2B shows the FBSG sub-guild, *i.e*., the right block in Fig. 1. Figure 3 shows the interactions between both the sub-guilds. While Fig. 2A,B are self-evident, more insights can be revealed by further analyzing the interactions between both the sub-guilds. In remaining part of this section, we focus on further exploring those interactions (in the forms of inter-species and inter-guilds) to complete the objective set for this article as introduced previously.

### FBA Guild—further analysis of the inter-species and inter-guild interactions

In the previous sub-section, we observed the two sub-guilds of the FBA guild, *i.e*., sub-guild ASG dominated by *Actinobacteria*, and sub-guild FBSG dominated by *Firmicutes* and its ally *Bacteroidetes*. Both sub-guilds compete with each other. The interactions (correlations) within each sub-guild are cooperative (positive), but the interactions between the two sub-guilds are competitive (negative).

To further explore the inter-species and inter-sub-guild interactions, we introduce the concept of ‘guard’ nodes. We define *guard* nodes of a sub-guild as nodes that have negative relationships with the nodes in another sub-guild, but with full positive relations with nodes within its own sub-guild. That is, guard nodes “guard against” their counterparts in another sub-guild, but are ‘friendly’ to their own sub-guild-members (*i.e*., their relationships with other sub-guild members are cooperative). Figure 3 shows the interactions between guard nodes from both ASG and FBSG sub-guilds. In Fig. 3, the left column exhibits the 14 guard nodes in the ASG, in which 7 species are from *Cornebacterium* genus, 2 species from *Actinomyces* genus, and the remaining 5 guard species from other small phyla but none from *Firmicutes* or *Bacteriodetes*. The right column of Fig. 3 displays the 16 guard nodes in the FBSG, in which 7 species are from *Firmicutes*, 4 from *Bacteriodetes*, 2 from *Actinobacteria*, and 3 from other phyla.

The taxonomic information *FBA*-guild is listed in Table 4, which is tabulated based on Figs. 1 and 3. In Table 4, nodes are classified into two types: one type is the guard node that is always ‘hostile’ (negative interactions) to its counterparts in another sub-guild but always ‘friendly’ to its *civilian* nodes within the same sub-guild; the other type is, what we called, civilian node who may be ‘friendly’ to any node in the whole *FBA* guild (or any sub-guild). The distinction between civilian nodes and guard nodes suggests the possible functional differentiations among the nodes in each sub-guild. It should be the differentiation that shape or even determine the *interactions* between two sub-guilds. Two types of nodes may play rather different roles in the interactions. The role (function) of *guard* nodes should be to protect their *home*-sub-guild against invasions from *foreign* guards, while they should never compete with any nodes in their homeland. Using an analogy, *civilian* nodes in both sub-guilds, although they have their own citizenships, can ‘friendly’ interact with any nodes regardless of their citizenship. Using another analogy, *FBA* guild is like a global village, where civilians may friendly trade with each other, but each sub-guild still preserves their military forces (guards) and ‘fight’ each other to keep order. This reminds us that, in the *FBA* triangle relationship, although *FB* (*Firmicutes & Bacteriodetes*) is united against A (*Actinobacteria*), the competition between *FB* & *A* only occurs in military sector and two sides (sub-guilds) cooperate with each other in civilian sectors.

**Table 4:** The classification of nodes as *guard* nodes and *civilian* nodes in each sub-guild.

Sub-Guild	Node Type	*Actinobacteria*	*Firmicutes*	*Bacteroidetes*	Others
**ASG** *(Actinobacteria-*dominant sub-guild)	Civilian Nodes	*Corynebacterium_767*		*Capnocytophaga_384*	
		*Corynebacterium_1*		*Capnocytophaga_252*	
		*Corynebacterium_413*			
		*Corynebacterium_291*			
		*Actinomyces_167*			
	Guard	*Corynebacterium_633*			*Bacteria_655*
	Nodes	*Corynebacterium_53*			*Fusobacterium_608*
		*Corynebacterium_414*			*Leptotrichia_615*
		*Corynebacterium_256*			*Neisseriaceae_683*
		*Corynebacterium_28*			*valvarum_597*
		*Corynebacterium_169*			
		*Corynebacterium_304*			
		*Actinomyces_500*			
		*Actinomyces_436*			
FBSG (*Firmicutes*-dominant sub-guild)	Civilian	*Rothia_93*	*Streptococcus_250*	*Prevotella_15*	*Bacteria_287*
	Nodes	*Rothia_492*	*Streptococcus_521*	*Bacteroidales_88*	*Bacteria_469*
		*Rothia_299*	*Veillonellaceae_681*	*Prevotella_272*	*Salinivibrio_60*
		*Actinomyces_426*	*Veillonellaceae_70*		
		*Actinomyces_204*	*Veillonella_87*		
			*Mogibacterium_78*		
			*Thermophilus_6*		
	Guard	*Rothia_64*	*Lactobacillales_246*	*Prevotella_21*	*Haemophilus_511*
	Nodes	*Rothia_456*	*Lachnospiraceae_89*	*Prevotella_443*	*Enterobacter_236*
			*Peptostreptococcus_4*	*pallens_179*	*Haemophilus_16*
			*Streptococcus_452*	*Prevotella_30*	
			*Veillonellaceae_445*		
			*Streptococcus_230*		
			*Oribacterium_135*		

Table 5 further lists all negative interactions (correlations) between both the sub-guilds of the FBA guild. Table 6 further lists the number of positive, negative and total interactions, respectively, between *Firmicutes, Bacteriodetes* and *Actinobacteria*. Table 6 also computed the P/N (positive to negative) ratio of links between the three phyla according to Ma (2017) P/N ratio approach^43^. Table 6 indicates that in the *F-B* alliance against *A*, *F* plays a larger role than *B* does, given that P/N ratio between *F* & *A* is approximately ½ that between *B* & *A* and small P/N ratio is resulted from larger number of competitive interactions (the denominator).

**Table 5:** Brief information on the negative interactions between the two sub-guilds of FBA guild*.

ASG (*Actinobacteria-*Dominant Sub-Guild)	FBSG (*Firmicutes*-Dominant Sub-Guild)	Correlation Coefficient (*R*)	*P*-value
***Actinobacteria***	***Actinobacteria***		
*Corynebacterium_414*	*Rothia_64*	−0.677	<0.001
*Corynebacterium_414*	*Rothia_456*	−0.646	<0.001
*Actinomyces_436*	*Rothia_456*	−0.685	<0.001
*Actinomyces_500*	*Rothia_64*	−0.614	<0.001
***Actinobacteria***	***Firmicutes***		
*Actinomyces_436*	*Streptococcus_452*	−0.696	<0.001
*Actinomyces_436*	*Oribacterium_135*	−0.662	<0.001
*Actinomyces_436*	*Streptococcus_230*	−0.629	<0.001
*Corynebacterium_304*	*Peptostreptococcus_4*	−0.613	<0.001
*Corynebacterium_414*	*Peptostreptococcus_4*	−0.643	<0.001
*Corynebacterium_414*	*Streptococcus_452*	−0.636	<0.001
*Corynebacterium_414*	*Streptococcus_230*	−0.653	<0.001
*Actinomyces_500*	*Lactobacillales_246*	−0.626	<0.001
*Actinomyces_500*	*Veillonellaceae_445*	−0.607	<0.001
*Corynebacterium_414*	*Lachnospiraceae_89*	−0.614	<0.001
*Corynebacterium_633*	*Streptococcus_230*	−0.608	<0.001
*Corynebacterium_256*	*Streptococcus_230*	−0.673	<0.001
*Corynebacterium_414*	*Oribacterium_135*	−0.606	0.001
***Actinobacteria***	***Bacteroidetes***		
*Actinomyces_500*	*Prevotella_443*	−0.629	<0.001
*Actinomyces_500*	*Prevotella_30*	−0.624	<0.001
*Corynebacterium_169*	*pallens_179*	−0.658	<0.001
*Corynebacterium_53*	*pallens_179*	−0.621	<0.001
*Corynebacterium_28*	*pallens_179*	−0.610	<0.001
*Corynebacterium_256*	*pallens_179*	−0.607	<0.001
***Actinobacteria***	***Others***		
*Corynebacterium_414*	*Haemophilus_16*	−0.652	<0.001
*Actinomyces_500*	*Haemophilus_511*	−0.624	<0.001
***Others***	***Bacteroidetes***		<0.001
*Neisseriaceae_683*	*pallens_179*	−0.620	<0.001
*Neisseriaceae_683*	*Prevotella_443*	−0.608	<0.001
*Neisseriaceae_683*	*Prevotella_21*	−0.604	0.001
*Bacteria_655*	*pallens_179*	−0.737	<0.001
*valvarum_597*	*pallens_179*	−0.657	<0.001
*Leptotrichia_615*	*pallens_179*	−0.647	<0.001
***Others***	***Firmicutes***		
*Neisseriaceae_683*	*Streptococcus_230*	−0.759	<0.001
*Neisseriaceae_683*	*Lactobacillales_246*	−0.605	0.001
*Neisseriaceae_683*	*Streptococcus_452*	−0.602	0.001
*valvarum_597*	*Streptococcus_452*	−0.618	<0.001
*valvarum_597*	*Streptococcus_230*	−0.615	<0.001
*Bacteria_655*	*Streptococcus_230*	−0.612	<0.001
***Others***	***Others***		
*Fusobacterium_608*	*Enterobacter_236*	−0.619	<0.001
*Neisseriaceae_683*	*Haemophilus_511*	−0.691	<0.001

*The negative interactions only occurred between the guard nodes distributed in two separate sub-guilds.

**Table 6:** The relationships (correlations) among *Actinobacteria*, *Firmicutes* and *Bacteroidetes* within the FBA guild, and their P/N (positive to negative) ratios*.

Phylum	Total Num. of Correlations	Positive Correlations	Negative Correlations	P/N (Positive to Negative) Ratios
*Actinobacteria vs. Firmicutes*	87	74	13	5.7
*Actinobacteria vs. Bacteroidetes*	63	57	6	9.5
*Firmicutes vs. Bacteroidetes*	85	85	0	Inf

*Total num. of correlations: The total number of correlation relationships between each pair of phyla in the guild.

Positive correlations: The number of positive correlation relationships between each pair of phyla in the guild.

Negative correlations: The number of negative correlation relationships between each pair of phyla in the guild.

P/N (positive to negative) ratios: The ratio of the number of positive correlations to that of negative correlations for each pair of phyla in the guild.

### FBA Guild—bring back the ‘ugly’ other phyla

**Figure 4:**
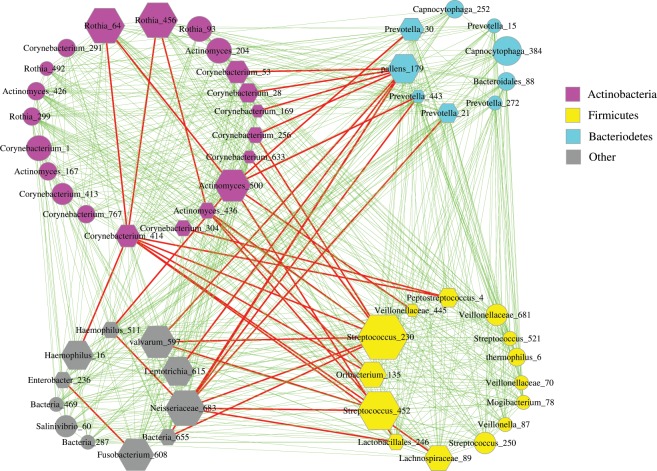
The relationships between *Actinobacteria*, *Firmicutes, Bacteroidetes* and the “other phyla” in the FBA guild. Symbols used: nodes in magenta—the OTUs of *Actinobacteria* phylum, nodes in yellow—the OTUs of *Firmicutes* phylum, nodes in cyan—the OTUs of *Bacteroidetes* phylum, nodes in gray— the OTUs of “other phyla”; edges in green—positive correlations, edges in red—negative correlations.

In previous sub-sections, we intentionally ignore the “others phyla” that do not belong to *Firmicutes*, *Bacteroidetes* and *Actinobacteria* to simplify the interpretation and presentation of our findings. Here we bring back “the others” in Fig. 4. In Fig. 4, rather than laying out the FBA cluster as a ‘bipartite’ network as in Figs. 2 and 3, the network was laid out as four blocks. Besides the three blocks of *Firmicutes*, *Bacteroidetes* and *Actinobacteria*, respectively, the “other phyla” occupied a fourth block (the left-down group, nodes in grey). First, the different layouts from Fig. 1 to Fig. 4 were made to facilitate the visual inspection of various facets of the network graph, and they, of course, influence neither the true cluster structure nor its interpretation. Second, most of the other group members belong to *Proteobacteria* and *Fusobacteria*, the two other core phyla Zaura *et al*. (2009) had already identified^24^. While the distributions of *Firmicutes*, *Bacteroidetes* and *Actinobacteria* are rather aggregated in the sense that they form strongly connected sub-clusters, the distributions of *Proteobacteria* and *Fusobacteria* are rather dispersed in the sense that they are distributed all over the place (all sub-clusters), and they do not dominate in any sub-clusters. Furthermore, “the others” do not seem to have a special or fixed pattern in their interactions with *Firmicutes*, *Bacteroidetes* and *Actinobacteria*. For example, while most interactions are cooperative, competitive relationships also exist. Using an analogy, we characterize “the others” as “nomads” of small “ethnic groups” in the FBA guild. Although further investigation on “the others” could be interesting, we believe the results should not affect the validity of the findings discussed in previous sections.

As a side note, we conducted similar examinations of other clusters detected with MCODE and listed in Table 2, but failed to find similarly interesting structures or interactions. Since majority of the core oral microbes (*i.e*., *Firmicutes, Proteobacteria, Actinobacteria, Bacteroidetes and Fusobacteria*) identified by Zaura *et al*. (2009) are contained in the *FBA* guild, which is the largest (also the strongest) cluster we detected, the failure should not be surprising^24^. Hence, the structure and species-interaction mechanism of *FBA* guild revealed in this article also represent those of core oral microbes.

### The mission of FBA guild in the oral microbiome—a new hypothesis

In previous sections, we have showed the structure and inter-species interactions within the FBA. Nevertheless, at this stage, we cannot fully explain the underlying mechanisms leading to this interesting triangular relationship, which requires experimental investigations beyond the scope of this article. Here, we propose a new hypothesis to explain the observed phenomenon, and hope to stimulate the further studies on this obviously rather important phenomenon.

First, both *Firmicutes* and *Bacteroidetes* have been the dominant players in the gut microbiome and have attracted extensive attentions in recent years, in particularly, their implications to obesity. The ratio of *Firmicutes* to *Bacteroidetes* (F/B) has been suggested as an index of the health of gut microbiome. Both the phyla are the most abundant taxa of gut microbiome, although the inter-individual variations are huge and their dynamics is rather dramatic^30,45–47^. For example, the F/B ratio could decrease from approximately 10.9 in middle-age adults to 0.6 in the elderly^45,48^. In the oral microbiome, Zaura *et al*. (2009, 2014, 2015) studies also suggested the dominance of both phyla, and contributed approximately 50% (36% *Firmicutes* and 12% *Bacteroidetes*) to the oral microbiome, and were two of the five major phyla in the oral microbes [the other three were: *Proteobacteria* (22%)*, Actinobacteria* (24%), *and Fusobacteria* (4%)]^23,24,33^. Since oral and gut environments are well connected, and bacteria may freely disperse but environment would select who can stay and who can only be by-passers or nomads^49,50^. Therefore, it can be expected that the oral and gut microbiomes should be of certain level of similarity. Therefore, the dominance of *F* & *B* in the oral environment can be expected, but we are puzzled by the fact that there were not any negative interactions between *F* & *B* (Table 5, Fig. 4). It might be just that the gut environment allows for the competition between the both because both *F* & *B* may be competing for the fermentation niche, one of the three metabolic niches (the other twos are sulfate reduction and methanogenesis) gut microbes compete for in the gut ecosystem^51^. Existing literature reveals that majority of species in *F* & *B* are involved in fermentation^51^. However, healthy oral environment is not a fermentation habitat in general, and therefore *F* & *B* lose the battle ground for competing, instead they may turn to cooperation (positive correlations), possibly forming an alliance against *Actinobacteria* as we discovered previously. But this leads to another question, which we try to answer below, why do *F* & *B* both do not like *Actinobacteria*?

Second, note that in the *Actinobacteria-*dominant sub-cluster, *Cornebacterium* and *Actinomyces* are the two primary genera. Existing literatures suggest that these two genera include some of the notorious pathogens, especially opportunistic pathogens. For example, *C. diphtheriae* causes diphtheria. Other pathogenic species in humans include: *C. amicolatum*, *C. striatum*, *C. jeikeium*, *C. urealyticum*, and *C. xerosis*^5,52–55^. Certain species of *Actinomyces* are known to be opportunistic pathogens, particularly, when the immune system of host is weak^56–61^. Of course, there are innocuous species in these genera, given that oral microbiome and its environment (host) usually live harmoniously and their interactions are cooperative in large^62–66^. We conjecture that the potentially suppression of *F-B* alliance against *A* exhibited by the *FBA* guild, as the primary component of core oral microbiome, should be important for maintaining a healthy oral microbiome and protect humans from many opportunistic infections.

A major limitation of this study is that the dataset used to reconstruct the oral microbiome network was published a decade ago, and the 29 samples were collected from three healthy individuals only (Zaura *et al*. 2009). Therefore, the findings from our reanalysis of the datasets should be validated with more extensive datasets in future. A primary motivation for us to publish our results was to demonstrate the potentially important application of the concept of ecological guild in microbiome studies. The dataset originally reported by Zaura *et al*. (2009), which we reanalyzed, offered us an excellent opportunity to pursue our objective because of its multi-site nature and high-quality sequencing experiments.

## Data Availability

The raw sequencing datasets were originally collected and published by Zaura *et al*. (2009). Detailed access information was available in “Zaura E, Keijser BJF, Huse SM, *et al*. (2009). Defining the healthy “core microbiome” of oral microbial communities. *BMC Microbiology*, 9: 259”.
